# yStreX: yeast stress expression database

**DOI:** 10.1093/database/bau068

**Published:** 2014-07-14

**Authors:** Kwanjeera Wanichthanarak, Intawat Nookaew, Dina Petranovic

**Affiliations:** ^1^Department of Chemical and Biological Engineering, Chalmers University of Technology, Gothenburg, Sweden and ^2^Comparative Genomics Group, Biosciences Division, Oak Ridge National Laboratory, Oak Ridge, TN 37831, USA

## Abstract

Over the past decade genome-wide expression analyses have been often used to study how expression of genes changes in response to various environmental stresses. Many of these studies (such as effects of oxygen concentration, temperature stress, low pH stress, osmotic stress, depletion or limitation of nutrients, addition of different chemical compounds, etc.) have been conducted in the unicellular Eukaryal model, yeast *Saccharomyces cerevisiae*. However, the lack of a unifying or integrated, bioinformatics platform that would permit efficient and rapid use of all these existing data remain an important issue. To facilitate research by exploiting existing transcription data in the field of yeast physiology, we have developed the yStreX database. It is an online repository of analyzed gene expression data from curated data sets from different studies that capture genome-wide transcriptional changes in response to diverse environmental transitions. The first aim of this online database is to facilitate comparison of cross-platform and cross-laboratory gene expression data. Additionally, we performed different expression analyses, meta-analyses and gene set enrichment analyses; and the results are also deposited in this database. Lastly, we constructed a user-friendly Web interface with interactive visualization to provide intuitive access and to display the queried data for users with no background in bioinformatics.

**Database URL:** http://www.ystrexdb.com

## Introduction

All organisms encounter various and dynamic changes in their environments. This also applies to single-cell organisms such as yeast *Saccharomyces cerevisiae*. The ability to respond appropriately to such variations is required for optimal growth, competitive fitness and cell survival. In case of yeast it was found that the stress-response strategies needed to deal with fluctuations in temperature, pH, nutrients, oxygen, induction of osmotic stress and the presence of various agents such as drugs and toxic compounds, rely extensively on genome-wide transcriptional changes (1–3). To study stress responses to diverse environmental changes and understand transcriptional regulations, DNA microarray technology has often been used (1, 2).

These genome-wide transcription studies have generated large and diverse data. The challenge is thus to create a unifying bioinformatics platform and resource where all relevant data from numerous sources are collected, making it possible to integrate and effectively query and exploit these data. Several databases such as ArrayExpress (4) and GEO (5) have been developed aiming to store and exchange gene expression data and associated information. However, if one would like to use the data deposited in these databases, one will still need to download and reanalyze the data to answer specific biological questions related to specific research questions. SPELL (6) and MEM (7) are examples of the databases that put an effort to maximize exploitation of gene expression data. Their scope lies specifically on gene coexpression searches over several data sets. Other databases such as Gene Expression Atlas (8) have also addressed this issue by providing features to query readily analyzed gene expression, but they do not include additional information such as enriched biological features of gene lists, which are useful for interpretation of the data.

Here we introduce yStreX, an online resource for yeast research with specific focus on stress responses. Here, we use the term ‘stress’ broadly and we apply it to different types of physical or chemical stresses, such as temperature or osmotic stress, nutrient stresses, oxygen limitation or addition of specific compounds that induce stress responses. We also included transcription data for aging; aging is not an external agent that induces a stress response, but rather an inherent property of the cell that can be influenced by environmental and genetic factors. However, an aging cell shows specific transcriptional responses that are related to stress responses, so we believe it is a valuable and appropriate addition to this database.

yStreX addresses the growing need to efficiently collect, utilize, distribute and query analyzed gene expression data from different original studies. Information from the gene expression analysis, including statistical values of genes and enriched biological features of gene lists under specific conditions can be easily explored through an intuitive user-friendly Web interface. In addition, creating the specific compendia of gene expression data allow us to conduct meta-analyses across different platforms and laboratories (i.e. combining gene expression data from independent but related studies), which can enhance statistical power, reliability of the results and generalization of conclusions (9). To demonstrate different features and illustrate how the database can be used in original research, we include two examples.

### Data preparation

Gene expression data were retrieved from ArrayExpress (4) and GEO (5). To enhance statistical reliability, data sets ‘with less than 2’ repeats/repetitions (biological or technical replicates, depending on what was available from the original published study) were discarded. The terms ‘stress’, ‘treat’ and ‘respond to’ were used to search and select putative data sets that were then manually curated. The selected data sets came from experiments that included one or more variations of environmental factor(s), such as physical or chemical factors mentioned above. We have retrieved 82 stress-related data sets from both Affymetrix gene chip and complementary DNA (cDNA) two-color platforms. For the Affymetrix platform, Piano R package (10) was used to preprocess the data. The microarray analysis functions in Piano include important functions in affy packages (11) including normalization, annotation and quality control. For the cDNA platform, normalized data were imported and manually evaluated with GEO2R (5).

**Figure 1.**
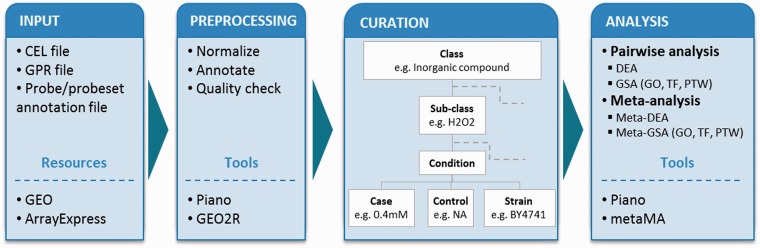
Analysis workflow. The diagram shows the workflow in the following steps: input, preprocessing, curation and statistical analyses. Tools and resources are also listed in the boxes. Microarray data sets from Affymetrix gene chip (CEL file) and cDNA two-color (GPR file) platforms were retrieved from GEO and ArrayExpress database together with probe/probeset annotation file. The data sets were preprocessed using Piano for Affymetrix and GEO2R for cDNA platform. Each data set was curated into defined experimental classes and subclasses, and it was considered in detail of experimental conditions (control values, case values, strains and type of repeats) based on its experimental details. Statistical analyses were performed including pairwise and meta-analysis. Both types of analyses were used to identify differentially expressed genes and enriched biological features: GO, TF and PTW.

To describe experimental conditions in a unified way (i.e. to allow for comparison between independent experimental setups, and for pooling the data in meta-analyses), we adapted the concepts of experimental factors (EFs) and EF values from Gene Expression Atlas (8). In our studies, we defined an experimental class as a major EF (e.g. C-source, aeration, inorganic compound) that is examined for gene expression changes and termed an experimental subclass as the specific experimental value of the class (e.g. within the class ‘inorganic compound’, the subclass can be ‘hydrogen peroxide’). Within the subclass, we further considered in detail for controls and cases (detailed or varied values) that are to be compared in differential expression analyses (DEA). We include strains’ description, and where applicable, we provide information about repeats/repetitions, as found in the original publication (Figure 1).

### Statistical analyses

Each gene expression data set was preprocessed, then mapped into classes, subclasses and curated as described above (see Supplementary Table S1 for the list of experimental conditions). Then for each experimental condition, we performed pairwise DEA to identify significantly differentially expressed genes. We then conducted the gene set analyses (GSA) on three levels of existing biological knowledge: gene ontology (GO) gene sets, transcription factor (TF) gene sets and pathway (PTW) gene sets (Figure 1). The GO term gene sets are based on GO annotations for yeast genes from the SGD database (http://www.yeastge nome.org). TF gene sets are based on TF–gene interaction from YEASTRACT (12) and (13), and the PTW gene sets are based on the genome-scale metabolic model iTO977 (14). The Piano R package was used for these analyses (10).

For DEA, the Piano uses the linear model fitting procedure of the limma package (15) to compute gene-level statistics (e.g. fold change, *t*-value and *P*-value) for each gene in each experimental condition. The *P*-values were adjusted for multiple testing with false discovery rate (FDR) at 5%. Then the adjusted *P*-values were used as the input of the Reporter algorithm (16), which was used for GSA to compile significant gene sets (gene set *P*-values) among GOs, TFs and PTWs in each experimental condition. In addition, fold changes were used to classify the directionality of gene expression changes, which can facilitate the interpretation of significant gene sets as described in (10).

Currently, there are 20 subclasses (Supplementary Table S1) that contain data from two or more independent but relevant studies. Meta-analysis was performed on each of those subclasses using metaMA R package (17). The metaMA uses a moderated effect size combination method, in which effect sizes were calculated from moderated t-test (i.e. we used limma) for each study and then were combined before testing for differentially expressed (DE) genes (FDR at 5%) of a specific subclass. Similar to DEA, the resulting test statistics or gene-level statistics (meta-z-scores) were the input for GSA, for computing significant gene sets and for directionality classification of the gene sets. With provided functions in the Piano, we ran multiple GSAs using five GSA methods (mean, median, sum, maxmean and page; see Varemo *et al.* (10) for details), which only accept z-scores as input and then combined GSA results to obtain gene set consensus scores. The Piano uses rank aggregation approaches to assess consensus scores, and in this study, the gene set consensus score was the median rank of each gene set given by different GSA methods (i.e. the lower the score, the higher rank given by most GSA methods). Results from all analyses including DEA and GSA for each condition, and meta-DEA and meta-GSA for each subclass were deposited in the database, and these results can be accessed through different functions on the Web interface.

### Database and Web interface

yStreX is a document-oriented NoSQL database, which is managed under MongoDB (http://www.mongodb.org). Data stored in the database are results from statistical analyses, and the data are deposited as sets of documents within different collections. We store a considerable amount of data and results from analyses, i.e. 121 conditions and 410 of statistical comparisons for ∼6000 genes produces about 2 million records that we store and manage in the database. The NoSQL database was chosen because it has emerged as a preferred database choice for big data applications, and it does not require a predefined schema in contrast to relational databases. This is beneficial for scaling out the database (splitting data across many servers) when the amount of data grows and cloud computing technology becomes increasingly available (18–20). The Web interface to explore the data was implemented on PHP (http://php.net) and JavaScript (http://en.wikipedia.org/wiki/JavaScript). The Shiny package (http://www.rstu dio.com/shiny) was integrated to present results from both GSAs and meta-GSAs in the form of heatmaps and data tables, which can be downloaded from the functions provided.

**Figure 2.**
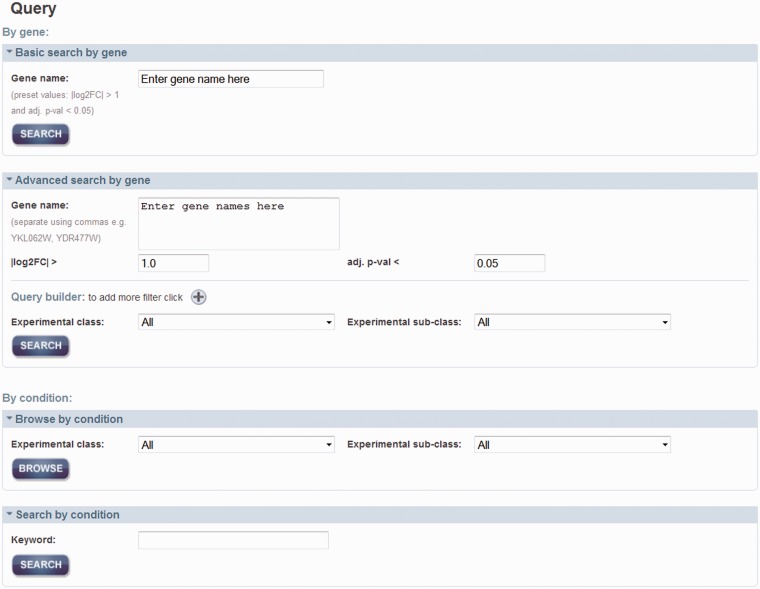
Query page. Two main approaches for query data are either by gene of interest or by condition of interest. Advanced search can be used to set additional properties that can add constrains in the query by gene name. Querying by a condition can be either by selection of the experimental classed or subclasses from the menu or by using a keyword.

As shown in Figure 2, there are two main approaches to query the data: by gene and by condition. By searching by a gene or a set of genes (i.e. systematic name, gene symbol or alias), one can retrieve a list of conditions where the gene(s)’ expression exhibited a significant change. For ‘Basic’ search by gene, it allows for searching by one gene with default properties; an absolute log2 fold change (|log2FC|) > 1 and an adjusted *P*-value (adj. *P*-val) < 0.05, which are set for simple and quick queries. Alternatively, it is possible to search by a set of genes with different properties such as log2FC, adj. *P*-val, experimental class and subclass to constrain the query in ‘Advanced’ search. Querying by a condition will retrieve a set of differentially expressed genes, as a result of the meta-analyses. This search can be accessed through a browsing option or searching by a text keyword.

## Results

Here we present two different examples of how to query yStreX and illustrate different features and practicality of the database.

### Example from differential gene expression analysis: expression of apoptosis genes during H_2_O_2_ treatment

Apoptosis is a type of programmed cell death (PCD) that has not only been extensively studied in mammalian cells but also in unicellular Eukarya such as yeast. PCD pathways can be induced by various environmental factors such as temperature stress, oxidative stress or induction with several types of drugs/chemicals. In this example, we queried yStreX with a set of 51 apoptosis-related genes that were retrieved from the yApoptosis database (21), and we searched for genes that significantly changed expression under oxidative stress induced by H_2_O_2_. This example presents features of the ‘Query by gene’, the retrieval of results from DEA (see Supplementary Figure S1A) and exemplifies what can be concluded by analyzing data from different studies. With the cutoff of |log2FC| > 2 and adj. *P*-val < 0.01, we found 14 apoptosis genes with significantly changed expression in 15 different studies where yeast was treated with H_2_O_2_. Of the 15 genes, 9 genes were significantly upregulated, and 5 genes were significantly downregulated (see Supplementary Table S2). These 15 studies use a different yeast strain, or different concentrations of H_2_O_2_ for induction, or the exposure time to H_2_O_2_. Because most of the apoptosis-related genes are regulated posttranscriptionally (e.g. proteolyic activation, phosphorylation and subcellular localization) (22), it is not surprising that less than half of apoptosis-related genes are differentially expressed in this apoptosis-inducing condition.

In this example, we show how we can use analyses of the existing data to find new results and propose hypotheses. For example, we found that OYE3 (coding for a NADPH oxidoreductase) was significantly upregulated in all 15 studies. It has previously been reported that OYE3 can promote H_2_O_2_-induced PCD in yeast (23). This could imply that ‘specifically’ transcriptional regulation of OYE3 might be an important step in H_2_O_2_-induced cell death. We also found that UBP10 (coding for a deubiquitylating protease) is significantly downregulated in all H_2_O_2_-inducing conditions. This could imply that there is another apoptosis gene that is ‘specifically’ transcriptionally regulated under oxidative stress. The yeast metacaspase has been shown to be activated upon H_2_O_2_ treatment (24); however, genes related to the caspase-dependent cell death pathway (e.g. DNM1 and MCD1) were not found to be significantly differently expressed in any study implying that this pathway is mostly posttranscriptionally regulated.

We show the possibility to retrieve information by browsing through the genes, in this case AIF1 (Apoptotic Inducing Factor 1). The ‘Gene summary’ page contains brief details about the selected gene and shows the list of conditions in which the expression of this gene was found to be significantly up- or downregulated, which is indicated by a red or green arrow, respectively. The list of presented conditions where the AIF1 gene is differentially expressed is arranged by the deceasing fold change (LogFC) (Supplementary Figure S1B). Yeast AIF1p is a caspase-independent cell death mediator that translocates from mitochondria to nucleus, leading to chromatic condensation and DNA degradation upon apoptotic induction (e.g. with addition of H_2_O_2_ or acetic acid or due to ageing) (25). There are 33 conditions in which the expression of AIF1 was found to be significantly changed (|log2FC| > 1 and adj. *P*-val < 0.01) (Supplementary Table S2). AIF1 was found to be downregulated only upon addition of rapamycin.

Lastly, we show the option to retrieve information through the conditions. The ‘Condition summary’ page comprises a brief description of the experimental condition (class and subclass of the experimental setup, case and control, yeast strain and the type of repeats if applicable) and a list of significant genes sorted by fold changes (Supplementary Figure S1B). In this example, yeast strain BY4742 was exposed to 0.4 mM H_2_O_2_. From comparison between the case (0.4 mM) and the control (untreated), there are 652 genes with significantly changed expression, within the specified cutoff |log2FC| > 1 and adj. *P*-val < 0.01). Genes (e.g. OYE3, CTT1, DDR2 and SOD2) having a role in oxidative stress response and cell death were strongly upregulated (log2FC > 2) (Supplementary Table S2). Moreover, well-known general stress response regulators such as MSN2, MSN4, XBP1 and GIS1 were identified from GSA, meaning that genes regulated by these TFs have significantly changed transcription. Enriched TF gene sets can be searched and downloaded as a heatmap or a tab-delimited text file through provided links (Supplementary Figure S1B).

### Example from the meta-analysis: rapamycin-induced gene expression

Inhibition of the TOR complex 1 (TORC1) by rapamycin resembles nutrient starvation, leading to changes in expression of genes shown to respond to starvation and stress (26, 27). In this example, we searched for common genes and consensus gene sets from independent studies that measured transcription profiles of genes under rapamycin treatment. This example illustrates how to retrieve information from meta-analysis (Supplementary Figure S2A). By using the ‘Query by condition’, we searched the ‘Rapamycin’ subclass, which contains data sets of 18 conditions from five related studies, done on two different platforms. Genes with significantly changed expression, identified by the meta-DEA (with FDR of 5%), were listed and sorted by the number of conditions that found upregulated genes. ‘Condition summary’ provided at the end of the page shows every condition combined in the meta-DEA and the gene-level statistics from DEA of the selected gene (Supplementary Figure S2B).

In this analysis, we found that genes related to autophagy, cytosol-to-vacuole targeting (CVT) pathway and heat shock response (e.g. ATG1, ATG3, ATG8, ATG14, APE1, AMS1, HSP26, HSP78 and HSP42) were upregulated, whereas genes involved in cell growth or encoding ribosome subunits (e.g. TEF4, RPL24, RRP9 and RPL12A) were downregulated. Subsequently, autophagy (GO:0006914), CVT pathway (GO:0032258) and cellular response to oxidative stress (GO:0034599) were found in the top rank and as the significant GO term gene sets from meta-GSA. Upon TORC1 inhibition, stress-response TFs are also identified. These TFs include MSN4, RIM15 and GIS1 meaning that genes under their regulation were found to be upregulated in all five studies. Besides this, metabolic adaptation was observed from both GO term and PTW gene sets, and this included tricarboxylic acid cycle, glycerol metabolism, glycogen metabolism and glutamate metabolism. This result was also confirmed by studies of essential roles of TORC1 during starvation, as described previously (28). Enriched gene sets (GO, TF or PTW) can be explored and downloaded as a heatmap or a tab-delimited text file through provided links in the Meta-GSA result (Supplementary Figure S2B).

## Conclusions and Perspectives

Over the past decade, several tools have been proposed for genome-wide gene expression analysis, as can be found in Bioconductor packages (http://www.bioconductor.org/). Typically, these tools carry out tasks to identify differentially expressed genes in a certain condition and infer correlation of differential expression information with existing biological knowledge (usually referred to as gene set enrichment analysis) (10).

We have developed yStreX, an online database to collect and distribute results of genome-wide gene expression analyses, including DEA and GSA. Based on the availability of gene expression data from the main repositories GEO and ArrayExpress, and our criteria about minimal number of repeats, we identified two main experimental platforms of interest: Affymetrix and the two-channel arrays. Because more studies aiming at transcriptional responses will be using RNA sequencing technology in the future, we envisage including gene expression data from this platform into yStreX. The database is specifically targeted for yeast data sets of experiments under several stress conditions, but it also includes transcriptional data of aging yeast, as its transcriptional response resembles a stress response, and direct comparison might be relevant for many members of the yeast research community.

A well-organized and user-friendly interface allows fast access and facilitates inference from the readily analyzed data, which is of practical use for experimental molecular biologists who do not wish to reanalyze data. This aspect is usually the limiting factor with databases that are just collecting and storing raw data, as they require the user to know in advance about the data and know how to carry out data analysis. However, such databases can be used in broader applications. To overcome this issue, we provided a link back to the original data allowing users to rapidly access raw data and carry out additional work (which might not be within the scope of the yStreX).

In addition, bringing together gene expression data and adapting the concepts of EFs make it possible to perform meta-analysis, which consequently increases statistical power, reliability and generalizability of the results, and makes use of the many experiments that have been carried out in different laboratories over the years. We demonstrated with two examples how the database can be readily used. The first shows the possibility to query many genes of interest concurrently (in this case, 51 apoptosis-related genes). Furthermore, we show that one can zoom-in on a gene of interest (e.g. AIF1) and retrieve the data of its transcriptional regulation under a stress of interest (e.g. oxidative stress by H_2_O_2_ induction). The second example shows the possibility to find common features, thanks to the meta-analysis approach, which combines data from many independent but related experiments.

Because of the limited number of high-throughput RNA sequencing data particularly for stress response studies in yeast, yStreX currently includes solely data sets from microarray-based assays. However, by using a document-oriented database, it is simple and flexible to scale out yStreX for including large and varied gene expression data in the near future.

An additional future challenge will be to uncover the transcriptional regulation of genes as the net result of multiple stimuli, as for now all results are based on comparisons of control versus one case (one stimulus or one testing condition). We are currently investigating statistical methods that would be appropriate for such situations. Moreover, in the future version of the database, we plan to include more features, more diverse download options and interactive pathway visualization that will continuously provide a useful service to the yeast research community.

## Supplementary Data

Supplementary data are available at *Database* Online.
